# Ligamentotaxis for complex calcaneal fractures using Joshi's external stabilization system

**DOI:** 10.4103/0019-5413.41858

**Published:** 2008

**Authors:** Ajai Singh, RN Srivastava, M Jah, Ashish Kumar

**Affiliations:** Department of Orthopedics, CSM Medical University, Lucknow, UP, India

**Keywords:** Calcaneal fracture, Joshi's external stabilization system, ligamentotaxis

## Abstract

**Background:**

Controversies exist in the literature regarding the management of complex fractures of the calcaneum. We evaluated a series of complex fractures of the calcaneum managed by ligamentotaxis using Joshi's external stabilization system (JESS) for its efficacy.

**Materials and Methods:**

Forty-five patients having complex (comminuted, intra-articular fracture with compromised soft tissue condition) fractures of the calcaneum, who were treated by external fixator (JESS) based on the principle of ligamentotaxis. The gradual distraction was done to bring the articular margins together to maintain both alpha and beta angles to near normal range. Thirteen (28.9%) patients underwent additional corticocancellous bone grafting with elevation of posterior facet. All patients were evaluated for their functional outcomes by American Orthopedic Foot and Ankle society (AOFAS) Score for the ankle and hind foot. Mean duration of follow-up was 20.5 months.

**Results:**

Forty-two (93.4%) of our patients did well with the ligamentotaxis. On evaluating final outcomes by AOFAS, approximately 71% of cases showed good results. Eleven patients (24.4%) complained of persistent heel pain in the long-term follow-up. Out of these, eight (17.8%) patients were those who had severe comminution with almost total loss of calcaneal height. The origin of heel pain was not the subtalar joint in all of these patients.

On long-term follow-up none of these patients suffered from such severe pain so as to compel them to change the nature of their activity.

**Conclusion:**

We conclude that ligamentotaxis by JESS provides a viable and user-friendly alternative method of management of these complex calcaneal fractures.

## INTRODUCTION

Magnuson stated that he “saw practically no fractures of the os calcis which did not result in significant disability of the foot”.^1^ Numerous controversies have surrounded the management of patients who have a calcaneal fracture.^2^ The primary source of disagreement has been the issue of whether better results are achieved with operative or non-operative treatment. The lack of a universal, consistent protocol for the subjective, objective and radiographic evaluation of these injuries has hampered the comparison of results. However, because different criteria for evaluation were used in most of these studies, meaningful comparison of the results has been impossible.^3^ To the best of our knowledge, there have been no reports of a large series of patients who were managed with different methods and assessed with a uniform evaluation system. Therefore, the use of a historical control group and comparison with different clinical series are necessary to resolve the controversies regarding treatment.^4^,^5^ The lack of a long-term follow-up studies, have led to disagreement regarding the important prognostic factors for patients who have a calcaneal fracture. Factors such as age of more than 50 years, existing subtalar osteoarthritis, and a decreased Bohler angle have been regarded as poor prognostic indicators by some authors but not by others.^3^,^6^–^8^ Various modes of treatment for the comminuted calcaneal fracture, including open reduction and fixation by plate, semi-invasive methods (Multiple K wires, Steinmen pin plaster, ligamentotaxis by ring fixator) and plaster, have been used by various workers and presented in recent literature.^7^,^8^ We conducted this study to evaluate the role of JESS in the management of fractures of the calcaneum, based on ligamentotaxis.

## MATERIALS AND METHODS

Forty-five patients of complex fractures of the calcaneum, presented between July 2003 to December 2005 were included in this prospective study. Any calcaneal fracture of less than three weeks duration, intra-articular comminution associated with widening of heel and with or without soft tissue compromise were included in the study. All calcaneal fractures of more than three weeks old, fractures associated with neurovascular deficit, associated with other ipsilateral fractures or with past history of injury or surgery on the same foot were excluded.

**Figure 1:**
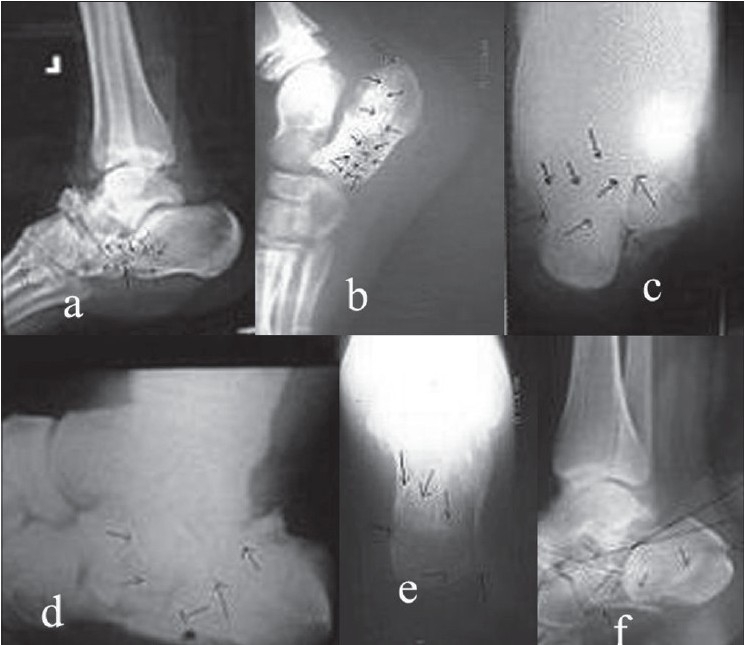
X-rays showing the fracture patterns of secondary fracture line (a) calcaneocuboid type of anterior secondary fracture lines, (b) plantar type of anterior secondary fracture lines (c) Type A of posterior secondary fracture lines pattern, (d) Type B of posterior secondary fracture lines pattern, (e) Type C of posterior secondary fracture lines pattern and (f) Type D of posterior secondary fracture lines pattern

The fracture classification of Essex-Lopresti^9^ was used to classify these fractures. We also observed for extent of secondary fracture lines extending from the primary shear line (as seen on axial and external oblique plain radiographs) to establish the comminution. The external oblique view for subtalar joint was performed by putting patient supine on table with knee at about 60 degrees flexion and limb rotated externally at 45 degrees with upright X-ray beam.^10^ Depending on these secondary lines in plain X-rays itself, we identified two broad patterns of these lines a) with anterior secondary fracture lines and b) with posterior secondary fracture lines. We identified two distinct patterns in the patients with anterior secondary fracture line [Figure 1a,b]: one entering the calcaneo-cuboid joint (calcaneo-cuboid type) and other entering the plantar surface of the calcaneum proximal to the calcaneo-cuboid joint (plantar type). Four distinct fracture patterns in posterior secondary fracture lines were observed [Figure 1c,d,e,f]; Type A: two-part shear fracture, Type B: fracture line exists below subtalar joint (Essex-Lopresti's depression/central depression type), Type C: fracture line exists at posterior cortex of tuberosity (Essex-Lopresti's tongue-shaped) and Type D: one with severely comminuted fracture. We observed that the extent of secondary lines correlated with comminution on plain X-rays itself.

Preoperative data were recorded related to associated injuries, the height from which the patient had fallen, type of occupation and later, course and duration of recovery. The length and weight of the patient was also recorded. The radiographic examination was done in detail which included lateral radiographs of both feet and anteroposterior radiographs of both ankles, axial radiographs of both hind feet, external oblique radiographs of both subtalar joints, and anteroposterior radiographs of both feet. The unaffected side was used to obtain control values for comparison of the measurements of subtalar incongruity, fibulo-calcaneal distance on the anteroposterior radiographs of the ankles, and of heel height, length of the Achilles-tendon fulcrum, angle of Guissane (Beta angle), talocalcaneal angle, and Bohler angle (Alpha angle) on the lateral radiographs.

### Operative procedure

**Figure 2:**
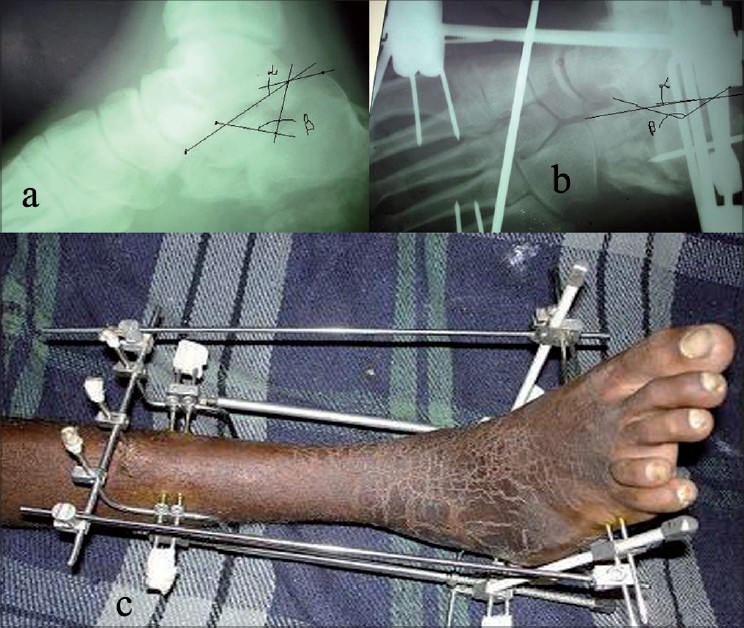
(a) Preoperative X-ray of the ankle (Lateral view) showing the type of fracture pattern and extent of comminution with significantly altered α and β angles of calcaneum. (b) Immediate postoperative X-ray of the ankle (Lateral view)showing fixator in position with on table partially corrected angles of calcaneum (c) Clinical photograph of the same shows the JESS fixator in position without any skin incision

All patients were given regional anesthesia and the frame was applied in supine position using power drill. Three tibial K-wires were passed from lateral to medial and both connected on each side of the leg by Z rod. The proximal arms of these Z rods were connected in front of the leg and to increase the stability of this frame one more K-wire was passed into the tibia in the anterio-posterior direction with precaution so as not to pierce the posterior tibial cortex. The distal arms of both Z rods were connected with each other on the posterior side of the leg. Two calcaneal K-wires passed medial to lateral and both connected with each other by one L rod on each side of the heel and the third K-wire passed from the posterior side of the heel into the calcaneum, which was then connected with a connecting rod with arms of both L rods. The first metatarsal K-wire passed from the base of fifth to first metatarsal (aimed to fix fifth, first and one of the middle metatarsal), while second K-wire from fifth to the fourth, just distal to the neck, and third from the first to the second metatarsal. After application of the frame, distractors were applied between the calcaneo-metatarsal and tibio-calcaneal holds on each side in the operation room itself. One tibio-metatarsal connector was applied to maintain plantigrade foot [Figure 2]. The angle of Gissane and Bohlar angles (i.e. height/length of calcaneum) were seen in the image intensifier. When elevation of the subtalar joint was required it was elevated either by using a percutaneous K-wire (*n* = 7) of 2.5 mm size (as joy stick) where a major bony articular fragment was available to elevate or by making a small window on the lateral wall of the calcaneum (*n* = 25) with cortico-cancellous autograft from iliac crest. In 13 cases with extensive comminution, it was difficult to elevate a single fragment hence distraction was delayed. Minimal distraction was done at the time of application of frame and continuous, gradual and regular distraction was delayed till the seventh postoperative day. This delay in distraction was aimed to get the advantage of soft callus distraction to form new bone. Meticuleous pin site care was ensured. All the link joints were tightened every week. Patients were advised for non-weight bearing walking with quadriceps drill and active mobilization of toes.

**Figure 3:**
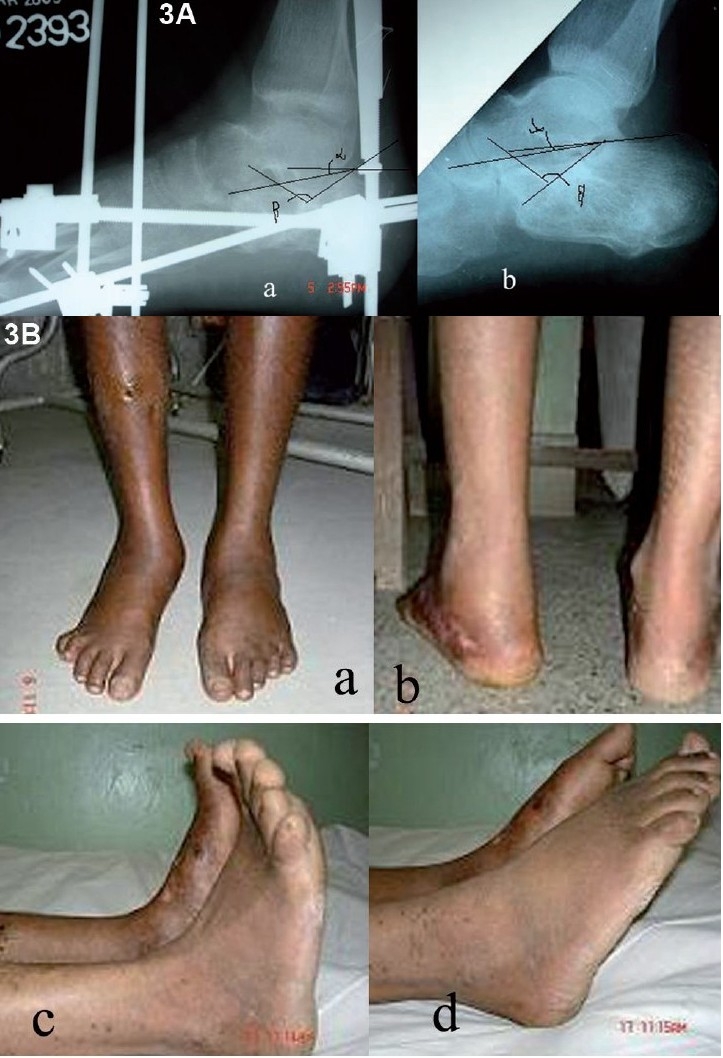
(A-B): (A) (a) X-ray of the ankle(lateral view) just before fixator removal showing evidence of bone healing with correction of altered calcaneal angles achieved by ligamentotaxis. (b) X-ray of the ankle of the same patient (lateral view) at one-year follow-up showing subtalar joint without any collapse of calcaneal angle correction, achieved by ligamentotaxis (B) Clinical Photograph of the same patient at final followup showing (a) full weight-bearing, (b) no significant clinical heel deformity, (c) and (d) good range of ankle movements achieved

Patients were taught to rotate the knobs of distractors by 360 degrees in a clockwise direction in four fractions i.e. 90 degrees every 6 h. End-point of distraction was judged radiologically (by regular follow-up X-rays done during distraction at weekly interval) till normal range of angle of Gissane (115-130 degrees) and Bohlar angle (20-40 degrees) was achieved [Figure 3a].

After achieving the normal range of the above angles the assembly was held in static position for a minimum of six weeks. The frame was then removed (early removal of fixators in only those patients, who did not wish to continue fixators due some reasons other than medical) and a patellar-tendon-bearing walking cast was applied for four to six weeks or if indicated, otherwise the assembly continued till the bony union was achieved. After this, patients were allowed to bear the weight gradually (non-weight-bearing to partial weight-bearing with support to unsupported full weight-bearing).

During follow-up, after the removal of fixators, patients had to undergo detailed physical examination to determine the location of the pain, if any. The sites of pain were located and graded in order of severity (most painful, second most painful, and so on). Locations of pain included subtalar region, lateral heel pain, the anterior tibio-talar region, the longitudinal arch, the metatarsal head, and the tarsal tunnel. The submalleolar width of the heel was determined with the use of Vernier calipers. After removal of fixator or final plaster, the patient was put on non-weight-bearing exercises followed by partial and, gradually full weight-bearing was allowed.

We evaluated our results using American Orthopedic Foot and Ankle Society (AOFAS) Score for the ankle and hind foot. In AOFAS, a maximum of 40 points are allocated for pain, 10 points for hind foot alignment and a maximum of 50 points are allocated to a variety of functions as determined by history and physical examination. Specifically, 10 points are allocated for activity level and aids used, five for walking distance, and five for ability to walk on uneven surfaces. Eight points are allocated for gait, eight points for ankle and hind foot stability, again eight for ankle motion and six for subtalar motion. The radiological angles thus achieved were measured and recorded at the time of removal of JESS and during the follow-up to observe the maintenance of these parameters. The first evaluation of the results was done on Day 0 i.e. the day of fixator/plaster removal. Then further regular evaluation of the results was done at six weeks, 12 weeks, six months and one year or at last follow-up.

## OBSERVATIONS AND RESULTS

Out of a total of 45 patients operated, 35 (77.8%) patients were male. The average age of these patients was 47.5 years (range 20-75 years). The average weight of the patients was 54.5 kg and average height was 162 cm. The right side was injured in 28 cases and the left side in 17 patients. Commonest mode of injury was fall from height in 33 (73.3%) patients. Twenty-nine (64.4%) patients presented to us within first week of injury. There were 20 cases of tongue type fracture pattern and the remaining 25 were of joint depression type of the Essex-Lopresti classification. Forty-two (93.4%) patients had fractures with posterior secondary fracture line and the remaining three (6.7%) patients had anterior secondary line. The calcaneo-cuboid type of anterior secondary line pattern was present in two (4.5%) cases and plantar type of anterior secondary line pattern was present in only one (2.5%) case. The posterior secondary line Type A pattern in two (4.5%) cases, depression/central depression (Type B) in 20 (44.5%) cases, tongue-shaped Type C pattern in 16 (36.5%) cases and Type D severely comminuted fracture line pattern in four (8.7%) cases. Twenty-eight (62.3%) patients showed significant preoperative widening of heel. The distraction was done for a mean period of 13 days (range 10-19 days). In 13 patients (28.9%) iliac crest cortico-cancellous bone grafts were used after elevation of subtalar joint at the time of the application of the JESS frame. Patients were followed up regularly. The mean duration of follow-up was 20.5 months (range, three to 38 months). Average time of union was 10.3 weeks (range 8.5 to 12.3 weeks).

In tongue-shaped fracture group the average pre-op α angle range was 13 degree (range 5-15 degrees) and the average β angle range was 137 degrees (range 125-145), which improved to average 27 degrees (20-30 degrees) and average 117 degrees (range 115-125 degrees) respectively. In joint depression type the preoperative α angle was, average 11 degrees (range 0-10 degrees) and β angle was average 139 degrees (range 135-145 degrees), which improved to average 25 degrees (20-30 degrees) and average 122 degrees (115-125 degrees) respectively. This improvement in α and β angles was statistically significant (*P* = 0.001) in both types of fractures. The angles thus achieved by the ligamentotaxis remain maintained by the time of removal of fixator or plaster. No patient presented a collapse of posterior facet of calcaneum or reversal of corrected angle, till the end of our follow-up.

Eight (17.8%) of our patients had superficial pin tract infection, which was controlled by suitable oral antibiotics and dressing. None of these infections warranted a premature change of wire-site or wire removal. Eleven (24.5%) patients had persistent heel pain during walking, mainly over the lateral side, out of which eight patients were those having severely comminuted fractures with near total loss of calcaneal height. But all were satisfied and none was so debilitating as to warrant a change in occupation. None reported any change in footwear due to heel broadening and all did well with the advice of modifications (to avoid prolonged standing, avoid walking without foot wears) in their day-to-day activities. In only five (45.5%) of these 11 patients, the source of pain was subtalar in origin, which was confirmed by infiltrating local anesthetic in the subtalar joint. Out of the remaining six patients presenting with heel pain on long-term follow-up, four (36.4%) had significant heel broadening and decreased fibulo-calcaneal distance in one (2.23%) patient and heel pad atrophy (*n* = 1, 2.23%) as a possible cause of heel pain.

On evaluation by AOFAS the final outcomes was good in 71% (*n* = 32) [Figure 3B] while 26.7% (*n* = 12) and 2.1% (*n* = 1) in fair and poor category respectively. Prognosis was explained to all these patients presenting with heel pain. They were managed conservatively (contrast foot bath, local irritant, if and when required) and were advised to avoid prolonged standing and walking without soft non-leather shoes. Long-acting local steroid infiltration was not given in any of these patients.

## DISCUSSION

The controversy concerning operative or non-operative treatment of calcaneal fracture has centered on the effect of an anatomical reduction on the clinical outcome.^11^ Any classification system depending on the comminution defined by the secondary fracture line pattern on plain X-rays, could not be found suitable to review our observations. We suggest that it may be relevant to study these secondary fracture lines on plain X-rays so that we can get a fair idea about the comminution.

The height of the heel was measured from the tip of the medial malleolus to the bottom of the heel pad. Antero-posterior radiograph of the ankle helps in measurement of the fibulocalcaneal distance between the tip of the lateral malleolus and the calcaneal shadow.^3^ The distance was decreased on the previously injured side. In this study, this decrease was found in 10 (22%) cases.

Our most important findings involved the relationship between the patho-anatomy and the results. A moderate increase in the clinically measured width of the heel was significantly associated with an unsatisfactory result.^13^ Similarly, there was a diminished fibulo-calcaneal distance in the patients who had an unsatisfactory result. An increased heel width and a decreased fibulo-calcaneal distance are caused by proximal and lateral displacement of the lateral wall or body of the calcaneus, or both. Given the relationship between the patho-anatomy and the results, a worse result would be expected in patients who have a more severe fracture. In the present study, about 22% had decreased fibulo-calcaneal distance while only 13.3% had significant heel broadening. Only < 3% had poor results as per AOFAS criteria. All the patients, who had a combination of heel broadening and decreased fibulo-calcaneal distance showed poor outcome in our study. Hammesfahr and Fleming reported better outcomes in patients who had a tongue-type fracture than those who had a central depression fracture.^14^ We found Essex-Lopresti's tongue-type fracture pattern in 22 (48.9%) of our patients. This study also demonstrated that patients who had a tongue-type fracture had the best prognosis.

Moderate comminution of central depression fractures worsened the prognosis. Extensively comminuted fractures were associated with the worst prognosis.^3^ Thus, careful classification of fractures on the basis of their appearance on plain radiographs can be very helpful in the prediction of clinical outcome. In the present study, we found central depression fracture pattern in 25 (51.1%) of our patients. All those patients who had poor outcome were having this fracture pattern.

According to our clinical evaluation, the subtalar joint was not the only source of pain in this series. Since our followup is short to develop subtalar osteoarthritis. Eleven (24.4%) of our patients presented with persistent heel pain in long-term follow-up. All these patients were subjected to this confirmation test by giving local anesthetic agent into the subtalar joint under aseptic technique. In only five (45.5%) of these 11 patients, the source of pain was found to be subtalar in origin. As in none of the patients this pain was so significant as to compel them to change their nature of activity, nothing active was done. They were explained about the prognosis and asked to remain in active follow-up. The other anatomical derangements increased heel width, heel-pad atrophy, and patho-anatomical alterations of the biomechanics of the foot--frequently lead to chronic pain, possibly more often than subtalar osteoarthrosis. Out of the remaining six patients presenting with heel pain on long-term follow-up, four (36.4%) had significant heel broadening and decreased fibulo-calcaneal distance was found in only one (2.23%) patient. Only one patient presented with heel pad atrophy as a possible cause of heel pain. We were not able to demonstrate subtalar osteoarthrosis in any of our patients, maybe due to a short follow-up. These findings were similar to the observations made by various workers in relation to the factors responsible for poor prognosis or suboptimal outcome.^2^,^3^,^7^,^10^,^12^

A recent review of the literature shows that various workers have used external fixators to manage these comminuted calcaneal fractures with open reduction and then maintenance by ligamentotaxis by the use of ring fixator.^13^–^15^ The principle of ligamentotaxis was used by these workers only to maintain the position of fragments with a tensioned/stretched soft tissue; and not for achieving the reduction. But we emphasize the importance of the application of the JESS distractor (based on the principle of ligamentotaxis) in such comminuted, intra-articular fractures because of its versatility, light weight, easy maneuverability of fragments even during distraction and the ease of elevating a depressed joint by making a small window under fluoroscopic guidance and filling the cavity with the bone graft. Following internal fixation by plate and screws, various workers had demonstrated the incidence of skin/soft tissue complications with or without deep infection to be as high as 12%.^9^,^16^ In our series we did not encounter any of these complications. Application of frame does not require any special instruments and is also safe even in less experienced hands. As the series is small and patients are still under follow-up it would be too early to say that it is the best available mode of treatment. Still, our results are encouraging and we could not find any significant major complication in this study.

We may conclude that ligamentotaxis by JESS provides a viable and user-friendly alternative method of management of these complex calcaneal fractures which may require further evaluation.
